# Molecular Monitoring of *Leiocassis longirostris* Using Species-Specific qPCR Assays from Environmental DNA

**DOI:** 10.3390/ani15233451

**Published:** 2025-11-29

**Authors:** Chang Gi Hong, Keun Yong Kim, Jung Soo Heo, Bo Hyung Choi, Ju Hwan Park, Seung Yong Kim, Seo Gyeong Yang

**Affiliations:** 1 Inland Fisheries Research Institute, National Institute of Fisheries Science, Geumsan-gun 32762, Republic of Korea; ckhong@korea.kr (C.G.H.); some1011@nate.com (S.Y.K.); 2 Bioinformatics Team, AquaGenTech Co., Ltd., Busan-si 48228, Republic of Koreadgyjs2@daum.net (J.S.H.)

**Keywords:** benthic ecology, freshwater fish, *Leiocassis longirostris*, environmental DNA (eDNA), qPCR, molecular monitoring, restocking, species-specific markers

## Abstract

*Leiocassis longirostris*, a large benthic catfish, has been classified as Regionally Extinct (RE) in South Korea since the 1970s. Assessing the success of ongoing restocking efforts in the Geum River is challenging due to limitations in traditional capture-based monitoring methods. We developed a highly sensitive, species-specific quantitative real-time PCR (qPCR) assay targeting the mitochondrial cytochrome *b* gene (*mt-cyb*) for this restocked fish. Assay validation confirmed high specificity against a panel of 25 co-occurring fish species, including 8 related siluriforms, and an excellent limit of detection of 10 copies per reaction. We applied this assay to environmental DNA (eDNA) extracted from paired water and sediment samples collected from 13 stations across the Geum River Basin. eDNA was detected at three water and five sediment stations. Notably, detection rates and average concentrations were consistently higher in a sediment sample (peaking at 1609.93 copies L^−1^), an observation consistent with the species’ benthic ecology. These findings provide the first molecular evidence that restocked *L. longirostris* may disperse and persist in the mid to lower reaches of the Geum River. This validated eDNA qPCR tool is essential for guiding the integrated monitoring and adaptive management strategies required to support the long-term recovery of this ecologically important species. This study is the first species-specific qPCR of the bagrid catfish in South Korea.

## Simple Summary

*Leiocassis longirostris* is a large bottom-dwelling fish that disappeared from South Korean rivers in the 1970s and is now listed as Regionally Extinct. To restore this species, restocking projects have been carried out in the Geum River, but it has been difficult to check whether the released fish are still surviving and spreading. This study aimed to develop a new, non-invasive way to monitor the species using environmental DNA, which detects tiny traces of genetic material left by fish in water or sediment. We created a highly sensitive DNA test that proved accurate even at very low concentrations. Using this test, we found genetic evidence of the species at several sites in the Geum River, particularly in sediment samples, which matches its bottom-dwelling nature. These findings provide the first molecular evidence that the restocked fish may live in the river. This approach provides an effective, non-invasive tool to monitor endangered freshwater species and supports long-term conservation.

## 1. Introduction

*Leiocassis longirostris*, commonly known as the longnose or Chinese longsnout catfish, is a large, benthic freshwater fish belonging to the family Bagridae (the order Siluriformes). This species is native to East Asia and has historically inhabited river systems in China and the Korean Peninsula [1,2]. It is an important commercial species in Chinese and Korean fisheries and aquaculture and typically found in large freshwater rivers with sandy or muddy substrates [2]. It has historically been reported in the Han, Imjin, and Geum River basins in South Korea. However, anthropogenic activities, including habitat loss, water pollution, and overfishing, have led to the classification of the species as “Regionally Extinct (RE)” in South Korea since the 1970s [1,3]. Restoration efforts were initiated in 2001 by introducing individuals weighing 1–1.5 kg and carrying out artificial seed production to reverse this decline. In 2008 and 2016, 5000 and 2000 artificially produced juveniles, respectively, were released into the Geum River basin. In 2017, an additional 200 mature individuals were released as prospective broodstock.

Until recently, the monitoring of *L. longirostrishas* was conducted only with traditional capture-based methods because molecular approaches, such as eDNA assays, were not yet available. Assessing the success of these restocking efforts using conventional monitoring methods (e.g., gill netting, electrofishing, and visual surveys) is challenging because of inherent limitations; these methods suffer low detection probabilities, can injure individuals and require expert morphological identification [4,5,6,7]. These constraints are particularly problematic for rare or endangered aquatic species, which typically occur at low densities and are difficult to sample effectively.

Environmental DNA (eDNA) offers a sensitive, non-invasive alternative for species detection by analyzing genetic material shed into aquatic environments [8,9,10,11,12]. This technique is now widely adopted for monitoring endangered and invasive aquatic species, usually using real-time PCR (qPCR) with species-specific primers. It provides high sensitivity and specificity, confirming their presence effectively, even in low-density populations, as successfully demonstrated in recent applications for rare or endangered fishes in both freshwater and marine systems [6,13,14].

Environmental DNA (eDNA) assays require careful development and validation to ensure reliable detection, particularly for rare or low-density freshwater species [10,13]. For benthic fishes, sediment can serve as an especially informative matrix because eDNA particles tend to accumulate and persist in bottom substrates more effectively than in the water column [11,15]. Previous eDNA applications for benthic or demersal fishes have shown that sediment sampling can complement water-based methods and improve detection sensitivity in lotic systems [6,16]. However, no eDNA assay has previously been developed for *Leiocassis longirostris*, making such background essential for interpreting the present study.

Ongoing monitoring of *L. longirostris* using traditional capture-based methods has yielded only sporadic data, insufficient for a robust assessment of its distribution. This study aimed to investigate the distribution of *L. longirostrisacross* in the river system by analyzing sediment-derived eDNA, taking advantage of the ecological characteristics of this benthic species. In this study, we (1) develop and validate species-specific qPCR markers for *L. longirostris* detection from eDNA extracted from water and sediment samples, and (2) apply the assay to evaluate its presence and distribution in the Geum River using this newly developed assay after stocking efforts. It is the first study on species-specific qPCR assay on bagrid catfish in South Korea.

## 2. Materials and Methods

### 2.1. Sampling and Genomic DNA (gDNA) Extraction of Fish Specimens

Eight specimens of *L. longirostris*, 2 adults (Llo-A001 and Llo-A002) introduced from China in 2000 and 6 of their offspring (Llo-R001–006), were collected from the National Institute of Fisheries Science of South Korea. Additionally, 25 non-target fish specimens were collected from South Korean rivers and lakes to ensure assay specificity. This specificity panel included 8 taxonomically related siluriform species (*Leiocassis ussuriensis*, *Liobagrus andersoni*, *Pseudobagrus brevicorpus*, *Pseudobagrus koreanus*, *Silurus asotus*, *Silurus microdorsalis*, *Tachysurus fulvidraco*, and *Tachysurus nitidus*) and 17 common freshwater species from various taxonomic groups (*Acheilognathus lanceolatus*, *Coreoperca herzi*, *Culter brevicauda*, *Cyprinus carpio*, *Erythroculter erythropterus*, *Gasterosteus aculeatus aculeatus*, *Lepomis macrochirus*, *Misgurnus anguillicaudatus*, *Monopterus albus*, *Odontobutis platycephala*, *Onchorhynchus mykiss*, *Orthrias nudus*, *Oryzias latipes*, *Plecoglossus altivelis*, *Repomucenus olidus*, *Rhynchocypris oxycephalus*, and *Zacco platypus*), also used in Kim et al. [14]. gDNA was extracted from a piece of pelvic fin tissue from each specimen using an APrep gDNA Tissue Kit (AP Bio Co., Ltd., Namyangju-si, Republic of Korea) and quantified/qualified using a NanoDrop One Spectrophotometer (Thermo Scientific Inc., Waltham, MA, USA).

### 2.2. DNA Sequencing

The mitochondrially encoded cytochrome *b* gene (*mt-cyb*) was amplified from the 8 *L. longirostris* specimens using a 20-μL PCR reaction in a ProFlex PCR System (Thermo Fisher Scientific Inc., Waltham, MA, USA) with degenerate primers [SIL-MT-14302f (5′-AACYAGGACYAATGACTTGA-3′) and SIL-MT-15591r (5′-ATYCTAGCTTTGGGAGTTAG-3′)] newly designed in this study. PCR was performed using the following cycling conditions in a ProFlex PCR System (Thermo Fisher Scientific Inc.): an initial denaturation at 95 °C for 5 min; 35 cycles of 95 °C for 30 s, 55 °C for 30 s, and 72 °C for 2 min; followed by a final extension at 72 °C for 5 min. PCR products were purified using the APrep PCR DNA kit (AP Bio Co., Ltd.) and directly sequenced on an Applied Biosystems 3730*xl* DNA Analyzer (Thermo Fisher Scientific Inc., Waltham, MA, USA). Their sequences are available in Supplementary Data S1 in FAST format.

### 2.3. Design of Primers and Probe

To design the species-specific qPCR primer pair and hydrolysis probe, the complete protein-coding *mt-cyb* sequences of the bagrid species were retrieved from the GenBank database of the National Center for Biotechnology Information (NCBI) (https://www.ncbi.nlm.nih.gov/; accessed on 2 September 2024) (Supplementary Table S1). The sequences, including the *L. longirostris* sequences generated in this study, were aligned using ClustalW, implemented in BioEdit 7.2.5, of which information is available in Supplementary Data S1 in FAST format. Species-specific nucleotide positions in *L. longirostris* were selected for primer and probe design. Oligonucleotide design criteria were primer length 18–28 nucleotides, 35–55% GC content, and melting temperature (*T*_m_) between 55 °C and 65 °C. The probe *T*_m_ was set at 4–7 °C higher than those of the primers. The length, GC content, *T*_m_, and secondary structures were predicted and optimized using Sequence Manipulation Suite ver. 2 [17].

### 2.4. Water and Sediment Sampling and eDNA Extraction

Water and sediment samples were collected on 8–9 March 2024, from 13 stations on the Geum River (Table 1). Before collecting water samples, water depth was measured using a depth sounder. For sites with depths of less than 3 m, water samples were collected from both the surface and bottom layers; for sites deeper than 3 m, they were taken from the surface, middle, and bottom layers. Water from each layer was collected using a 5 L sterilized water sampler, pooled into a sterile vinyl bag, and then subsampled into a 1 L sterilized sampling bottle. The water sampler was cleaned with sterile distilled water followed by 99% ethanol before and after use. The depths at sampling sites varied by location, ranging from 2 to 11 m.

Sediment samples were collected using a grab (20 × 20 cm). Approximately 20 g of sediments were subsampled from top 15 cm. The collected sediment was transferred into 2 L sterilized sampling bottles. The grab was cleaned with sterile distilled water followed by 99% ethanol before and after use.

The samples were stored in a cooled light-protected icebox and transported to the laboratory. For the water samples, 1 L was immediately vacuum-filtered through a glass microfiber filter (Grade GF/F circles, 47 mm, Whatman, Marlborough, MA, USA). Filters were stored at −20 °C until ready to use, broken up using a Bead Ruptor 12 (OMNI International, Kennesaw, GA, USA), and eDNA from each filter was extracted using the DNeasy Blood & Tissue kit (Qiagen, Hilden, Germany), eluted in 50 μL. For sediment samples, 10 g (wet weight) was broken up using the Bead Ruptor 12, and eDNA was extracted using the DNeasy PowerMax Soil Kit (Qiagen), eluted in 50 μL.

### 2.5. qPCR Analysis

qPCR was performed in a 20-μL reaction in triplicate using GoTaq Probe qPCR Master Mix (Promega, Madison, WI, USA). The reaction contained 1 μL of eDNA template, 0.2 μM of the forward and reverse primers [Llo-CYB-0535f (5′-GCATTTCACTTTCTACTTCCATTC-3′) and Llo-CYB-0741r (5′-TCTGGGTCTCCTAGCAGA-3′)], and the hydrolysis probe [Llo-CYB-0654p (5′-FAM-CTTCCATCCATACTTCTCCTATAAAGACA-BHQ1-3′)]. Assays were run on a QuantStudio 5 Real-Time PCR System (Thermo Fisher Scientific) with the following thermal profile: initial activation at 95 °C for 2 min; followed by 50 cycles of 95 °C for 15 s and annealing/extension at 63 °C for 45 s. *L. longirostris* gDNA (2 ng μL^−1^) and its culture water were used as positive controls, and sterile distilled water was used as a negative control. Since 1 μL of the extracted eDNA was used per qPCR reaction, the copy number obtained from the standard curve was multiplied by the elution volume (50 μL) to convert reaction-level copy numbers into copies per 1 L of water or 10 g of sediment.

The assay sensitivity was tested against a 10-fold serial dilution of plasmid DNA in 10^9^ copies per reaction (rxn^−1^), containing the *mt-cyb* target fragment, in triplicate. The results were used to generate standard calibration curves for the eDNA quantification. Specificity was confirmed against a panel of 8 siluriform species and 17 common freshwater fish species.

For qPCR validation, a 10-fold serial dilution of plasmid DNA ranging from 1 to 10^9^ copies per reaction was used to construct the standard curve. The curve consisted of seven dilution points and showed strong linearity (R^2^ = 0.998) with an amplification efficiency of 88.2%. The limit of detection (LOD) was defined as the lowest dilution that consistently produced amplification across triplicate reactions (10 copies per reaction), and the limit of quantification (LOQ) was set at 100 copies per reaction, which generated stable and precise quantification cycle (C_q_) values. Plasmid copy numbers for each dilution point were calculated from the supplier-provided DNA concentration information.

To monitor potential contamination, sterile distilled water was included as a negative control in every qPCR run, and no amplification was observed throughout the analyses. Although field blanks, filtration blanks, and extraction blanks were not incorporated in this study, all sampling and laboratory procedures were performed using sterilized single-use sampling bottles, filtration units, PCR tubes, and filter tips to minimize contamination risk. All eDNA extractions and qPCR setup steps were conducted in physically separated work areas dedicated to pre-PCR preparation.

## 3. Results

### 3.1. Sequencing and Design of Primers and Probe

Sequencing of the *mt-cyb* gene region from *L. longirostris* specimens in this study revealed that the 2 parents showed 100% sequence similarity, indicating very low genetic diversity within the broodstock population used for the restocking program in South Korea. A consensus sequence was generated to design a species-specific qPCR marker that enhanced the representation across all *L. longirostris* sequences.

**Figure 1:**
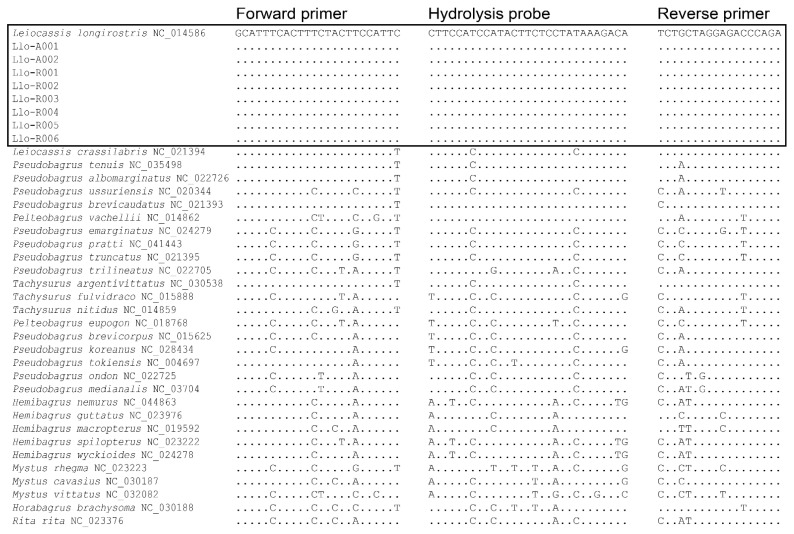
Binding sites and alignment matrix of forward and reverse primers and hydrolysis probe specific to *Leiocassis longirostris* from the mitochondrially encoded cytochrome *b* gene region. Oligonucleotid sequence of the reverse primer should be reverse complement from the binding site. the Dots represent the identical oligonucleotide bases of *L. longirostris* with the other species belonging to the family Bagridae in the order Siluriformes.

The final qPCR assay included forward primer (Llo-CYB-0535f), reverse primer (Llo-CYB-0741r), and hydrolysis probe (Llo-CYB-0654p), labeled with a reporter (FAM at the 5′-end) and a quencher (BHQ1 at the 3′-end) (Figure 1). This combination amplifies a 224 bp amplicon and is unique to *L. longirostris* among the other siluriform species examined.

### 3.2. Sensitivity and Specificity Tests

**Figure 2:**
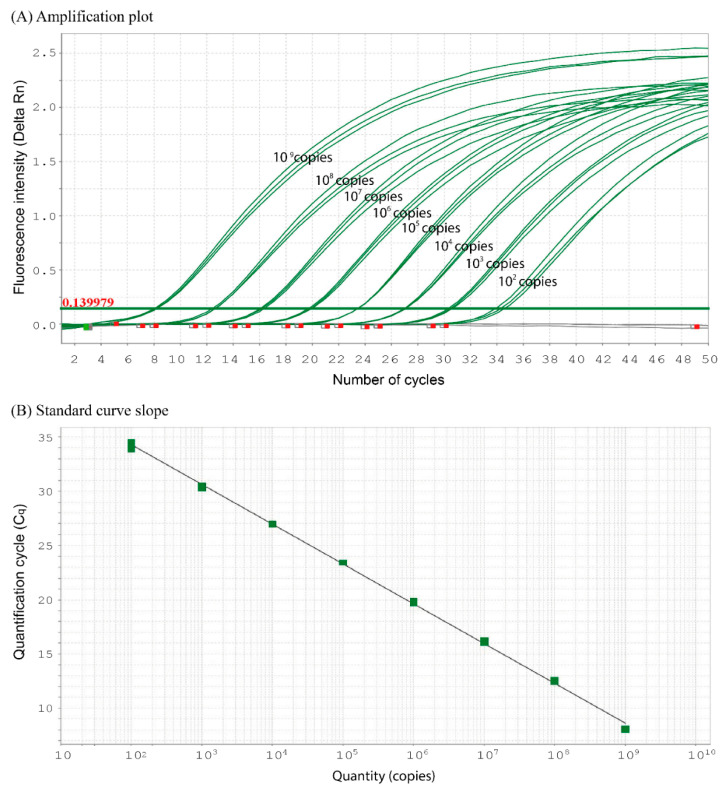
A sensitivity test of the primers and hydrolysis probe, newly designed in this study, specific to *Leiocassis longirostris*. (**A**) Amplification plot showing a serial dilution of plasmid DNA (1–10^9^ copies rxn^−1^) and (**B**) standard-curve slope producing a linear regression.

The positive control (gDNA at 20 ng μL^−1^) yielded a clear positive signal with a C_q_ value of 21.50. A sensitivity test using serial dilutions of plasmid DNA (Figure 2) demonstrated an inverse linear relationship between the target concentration and the C_q_ values. The assay achieved a limit of detection (LOD) as low as 10 copies rxn^−1^ (C_q_ = 37.50 ± 1.28) and a limit of quantification (LOQ) of 100 copies rxn^−1^.

A C_q_ value of 34.19 ± 0.34 was established as the lowest detection limit for acceptable precision and accuracy. The standard curve produced the following linear regression equation.

y = −3.64x + 41.424 (*r*^2^ = 0.998, efficiency = 88.236%)

This equation was used to detect and quantify eDNA in environmental samples.

**Figure 3:**
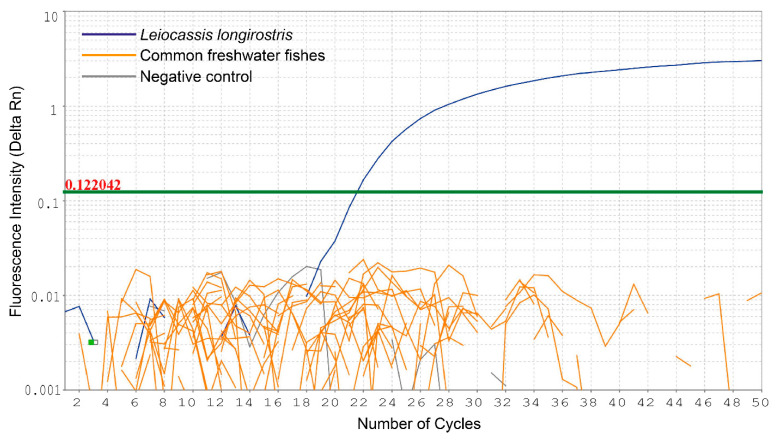
A specificity test of the primers and hydrolysis probe specific to *Leiocassis longirostris* developed in this study against other siluriform species and freshwater fish species commonly found in rivers and lakes in South Korea.

The specificity test confirmed that the qPCR marker successfully amplified DNA from the *L. longirostris* specimen, but failed to produce a signal against the 8 other siluriform species and the 17 common freshwater species (Figure 3).

### 3.3. qPCR Assay of Environmental Samples

Analysis of eDNA from the Geum River revealed the presence of *L. longirostris* eDNA at a subset of the surveyed sites. The eDNA of the water samples (Table 2) was detected in water samples at three of the 13 stations. St. 8 (Imcheon-myeon) showed the highest average concentration (1379.30 copies L^−1^) with C_q_ values between 35.04 and 38.06 in all replicates. High variability among the replicates (standard deviation = 1499.18) was also observed. St. 11 (Napo-myeon) showed consistent detection in all replicates (C_q_: 36.12–36.85), with an average concentration of 1375.30 copies L^−1^. St. 13 (Seongsan-myeon) had a single positive replicate (C_q_ = 36.17), resulting in a concentration of 1530.2 copies L^−1^. This single-replicate signal was therefore treated as a tentative, low-confidence detection. The remaining ten water stations showed no detectable signals (ND).

eDNA was detected in the sediment samples from five stations (Table 3). St. 10 yielded the highest average concentration (1609.93 copies L^−1^, C_q_: 35.08–35.93) in two replicates. Sts. 4, 12, and 13 also showed strong signals with average concentrations of 1363.60, 1098.80, and 1297.77 copies L^−1^, respectively. St. 3 presented low concentration (546.03 copies L^−1^). The remaining 8 sediment stations showed no amplification in the sediment samples.

Most of the positive signals were detected at C_q_ values of 35–38, which lie near the assay’s LOD and are therefore associated with higher variability and a greater likelihood of stochastic amplification. Such high-C_q_ detections also carry a greater risk of false positives, as low-template amplification near the LOD can be strongly affected by stochastic effects.

## 4. Discussion

In this study, we sequenced the *mt-cyb* gene region from *L. longirostris* specimens and showed that the 2 parents as well as 6 offspring showed 100% sequence similarity. This result indicates low genetic diversity within the broodstock introduced from China and used for the restocking program in South Korea. In the future study, the breeding program by introducing broodstock after considering their genetic diversity will help establish healthy and stable population for fisheries resource enhancement.

**Figure 4:**
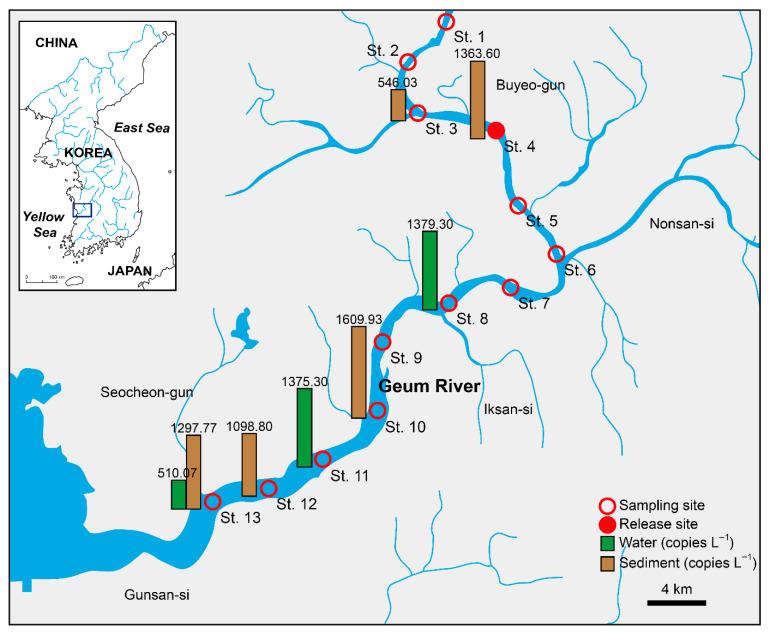
Average environmental DNA (eDNA) concentrations (copies L^−1^) of *Leiocassis longirostris* for the water and sediment samples collected from 13 stations in the Geum River basin, quantified using the real-time PCR assay.

We developed and validated a species-specific qPCR assay targeting *mt-cyb* to detect *L. longirostris* eDNA, and applied it to paired water and sediment samples from the Geum River. The assay revealed spatially restricted but repeated detection at three water (Sts. 8, 11, and 13), and five sediment stations (Sts. 3, 4, 10, 12, and 13), with the highest average concentration observed in a sediment sample at the downstream site (e.g., St. 10: 1609.93 copies L^−1^). The overall detection pattern, particularly at downstream stations (Sts. 8, 10–13), suggested the potential dispersal or downstream transport of eDNA molecules. These patterns are consistent with the localized presence of the species in the mid- to lower-river reaches following past releases. The distribution and magnitude of detections summarized in Table 2 and Table 3 (see also Figure 4) provide the first river-wide molecular evidence of post-stocking occurrence of this regionally extinct taxon in the lower Geum River, South Korea.

Differences in eDNA detectability between sediments and the water column are well documented in lotic systems and arise from distinct physical, chemical, and biological processes that govern DNA transport, deposition, and persistence. For benthic fishes, eDNA released near the streambed can readily adsorb to organic matter or mineral particles, allowing it to remain in sediments for extended periods and resulting in local accumulation [18,19]. However, sediment-derived eDNA often exhibits pronounced spatial heterogeneity because its retention is strongly influenced by microscale habitat variability, sediment particle-size composition, hydrological shear stress, and localized microbial degradation. Consequently, even within a single site, sediment eDNA can show irregular or inconsistent detection patterns [20,21]. In contrast, eDNA in the water column is rapidly mixed and transported downstream by flow velocity and turbulence [22,23]. When eDNA has recently entered from upstream sources or has not yet settled into the sediment, detection may occur exclusively in the water samples [18,24]. The contrasting detection patterns observed at Stations 8 and 11 likely reflect recent upstream transport of eDNA during the sampling period, whereas the low-level detection at Station 13 may be attributed to upstream dilution or reduced local input. To verify these mechanisms, additional investigations across seasons, hydrological conditions (e.g., pre- and post-rainfall), and variable flow regimes will be necessary.

Overall, our data showed wide ranges of the average and standard deviation values of eDNA copy numbers. High variability among replicates, including large standard deviations, is common in lotic eDNA samples near the detection limit due to heterogeneous particle distribution and stochastic capture [11]. The exceptionally high standard deviation observed at St. 8 likely reflects strong local turbulence and highly patchy eDNA distribution at this site, where template concentrations remained close to the LOQ and amplified replicate-to-replicate stochasticity. Because most detections occurred at high C_q_ values close to the assay’s LOD, these signals should be interpreted cautiously, as low-level amplification is more susceptible to stochastic effects and may carry a higher false-positive risk. Stations with only one of three replicates positive were therefore treated as tentative, low-confidence detections, reflecting the limited reliability of signals near the quantification threshold.

eDNA detection rates and concentrations were generally higher in the sediments than in the water column, which is in accordance with the benthic and demersal ecology of *L. longirostris* and its foraging in small fish and shrimp [2,25,26]. Benthic residence and site fidelity can promote the deposition and retention of cellular material in bottom substrates, increasing the probability of sediment detection relative to water detection at the surface or mid-water. Downstream positives in both matrices (water: Sts. 8, 11, and 13; sediment: Sts. 10, 12, and 13) suggested dispersal from the release area (St. 4) and occupancy of suitable habitats in the lower Geum River, consistent with expectations for species known to use lower river and estuarine environments [27,28].

The qPCR marker newly developed in this study demonstrated high analytical sensitivity (limit of detection, 10 copies rxn^−1^; limit of quantification, 100 copies rxn^−1^) and target specificity against a multi-species freshwater panel, supporting its suitability for the surveillance of low-density populations. These performance characteristics are consistent with those of previous studies, showing that species-specific qPCR can outperform traditional sampling methods for rare or elusive taxa [6,14]. In our data, some water samples exhibited replicate variability (e.g., St. 8), a common feature of lotic eDNA sampling driven by patchy shedding, turbulent mixing, and stochastic particle capture [11,16].

As with all eDNA surveys, eDNA detection indicated that *L. longirostris* was present at or downstream of the sampling locations; no definitive evidence of live individuals was observed at the time of sampling. Transport, dilution, and resuspension from substrates can move eDNA away from sources in rivers, and persistence times can differ markedly according to matrix type and environmental conditions [10,15,29]. Single-replicate detection at Sts. 3, 12, and 13 in both the water and sediment matrices merits cautious interpretation near the quantification threshold of the assay, although convergence with the strong sediment signals at Sts. 12 and 13 increases the confidence in the local occurrence. Such 1-of-3 replicate detections were considered low-confidence signals, reflecting the inherent stochasticity of amplifications near the assay’s detection limit. In addition, low-level detections near the assay’s LOD inherently carry a higher risk of false positives due to stochastic amplification or trace environmental contamination; however, the absence of amplification in all negative controls and the spatial consistency of detections across adjacent stations suggest that these signals are unlikely to represent spurious positives. Future analyses using occupancy modeling with explicit detection probabilities and covariates (e.g., discharge, turbidity, and substrate) would strengthen the inferences on site occupancy and distribution dynamics.

The detections at multiple, mainly downstream stations (from St. 4) suggest that the released *L. longirostris* are likely dispersed and may persist within suitable reaches of the Geum River. eDNA monitoring should be integrated with (i) seasonal resampling to capture phenology in shedding and hydrology, (ii) targeted conventional sampling (e.g., nets) at eDNA-positive hotspots to obtain morphological vouchers, and (iii) population genetic assays to assess the effective size and admixture relative to the broodstock to evaluate whether a self-sustaining population has been reestablished [30,31,32]. This tiered design follows the current best practices in conservation monitoring, in which eDNA is used to guide and prioritize more invasive follow-up sampling.

In summary, our species-specific qPCR assay provides a sensitive, noninvasive tool for detecting *L. longirostris* eDNA in the Geum River, yielding operationally useful distributional signals that complement conventional methods. Based on these findings, we recommend (1) temporal replication across seasons and hydrological conditions; (2) paired depth-integrated water sampling with standardized sediment coring to reduce matrix-driven bias; (3) incorporation of site-level environmental data to improve the robustness of distribution estimates; and (4) expansion to droplet digital PCR or multiplex qPCR for confirmatory and cost-efficient multi-species tracking in shared habitats. These results support continued hypothesis-driven monitoring to verify population establishment and to provide management with actionable triggers for habitat protection or additional releases, ultimately guiding the adaptive management of this culturally and ecologically significant species.

## 5. Conclusions

This study successfully developed and validated a species-specific qPCR assay to detect *L. longirostris* eDNA from freshwater systems. The assay demonstrated high sensitivity and specificity, with no cross-reactivity to non-target species and a detection limit as low as 10 copies rxn^−1^. Application of the assay to water and sediment samples from 13 sites along the Geum River revealed positive detections at 3 water stations and 5 sediment stations, including both the release site of artificially produced juveniles and multiple downstream locations.

The frequency and concentration of *L. longirostris* eDNA were higher in sediment samples than water samples. Our result partially reflects the benthic ecological characteristics of *L. longirostris*, a bottom-dwelling species. Detection in multiple downstream sites suggests that released individuals have successfully dispersed and may suggest successful reestablishment within the river system, providing molecular evidence supporting the potential for population reestablishment. In the future study, the presence of live individuals, reproduction, or self-sustaining populations should be confirmed.

These findings demonstrate the value of eDNA-based qPCR as a non-invasive, sensitive, and efficient tool for monitoring rare or regionally extinct species, especially when conventional survey methods are limited. Continued application of this method, combined with seasonal sampling, habitat modeling, and additional ecological or genetic analyses, will be essential for evaluating the long-term success of restoration efforts and guiding future conservation management of *L. longirostris*.

## Figures and Tables

**Table 1:** Information on sampling sites of water and sediment samples for the molecular monitoring of *Leiocassis longirostris*, located in the Geum River in South Korea.

Station	GPS Coordinate	Location	Water Depth (m)
St. 1	36°18′58″ N 126°55′56″ E	Buyeo-eup, Buyeo-gun, Chungcheongnam-do	2.5
St. 2	36°17′18″ N 126°54′09″ E	Gyuam-myeon, Buyeo-gun, Chungcheongnam-do	4.3
St. 3	36°15′11″ N 126°54′24″ E	Gyuam-myeon, Buyeo-gun, Chungcheongnam-do	6.6
St. 4	36°14′34″ N 126°57′35″ E	Buyeo-eup, Buyeo-gun, Chungcheongnam-do	2.6
St. 5	36°12′13″ N 126°58′14″ E	Sedo-myeon, Buyeo-gun, Chungcheongnam-do	5.1
St. 6	36°09′59″ N 127°00′16″ E	Ganggyeong-eup, Nonsan-si, Chungcheongnam-do	3.6
St. 7	36°08′58″ N 126°58′15″ E	Yongan-myeon, Iksan-si, Jeonbuk-do	10.7
St. 8	36°08′28″ N 126°55′34″ E	Imcheon-myeon, Buyeo-gun, Chungcheongnam-do	2.3
St. 9	36°07′31″ N 126°52′47″ E	Ungpo-myeon, Iksan-si, Jeonbuk-do	3.0
St. 10	36°04′48″ N 126°52′23″ E	Ungpo-myeon, Iksan-si, Jeonbuk-do	3.6
St. 11	36°02′57″ N 126°50′14″ E	Napo-myeon, Gunsan-si, Jeonbuk-do	3.4
St. 12	36°01′51″ N 126°47′46″ E	Napo-myeon, Gunsan-si, Jeonbuk-do	7.7
St. 13	36°01′35″ N 126°45′43″ E	Seongsan-myeon, Gunsan-si, Jeonbuk-do	5.0

**Table 2:** Quantification of environmental DNA (eDNA) of the water samples from the Geum River of South Korea using real-time PCR analysis with the primers and hydrolysis probe specific to *Leiocassis longirostris*.

Station	Quantification Cycle (C_q_) Value			Total Volume Equivalent (Copies L^−1^)			Average	Standard Deviation
Replicate	1	2	3	1	2	3		
St. 1	ND	ND	ND	0.0	0.0	0.0	0.00	0.00
St. 2	ND	ND	ND	0.0	0.0	0.0	0.00	0.00
St. 3	ND	ND	ND	0.0	0.0	0.0	0.00	0.00
St. 4	ND	ND	ND	0.0	0.0	0.0	0.00	0.00
St. 5	ND	ND	ND	0.0	0.0	0.0	0.00	0.00
St. 6	ND	ND	ND	0.0	0.0	0.0	0.00	0.00
St. 7	ND	ND	ND	0.0	0.0	0.0	0.00	0.00
St. 8	37.78	38.06	35.04	559.9	468.4	3109.6	1379.30	1499.18
St. 9	ND	ND	ND	0.0	0.0	0.0	0.00	0.00
St. 10	ND	ND	ND	0.0	0.0	0.0	0.00	0.00
St. 11	36.85	36.12	36.16	1002.3	1577.7	1545.9	1375.30	323.42
St. 12	ND	ND	ND	0.0	0.0	0.0	0.00	0.00
St. 13	ND	36.17	ND	0.0	1530.2	0.0	510.07	883.46

ND: not detected.

**Table 3:** Quantification of environmental DNA (eDNA) of the sediment samples from the Geum River of South Korea using real-time PCR analysis with the primers and hydrolysis probe specific to *Leiocassis longirostris*.

Station	Quantification Cycle (C_q_) Value			Total Volume Equivalent (Copies L^−1^)			Average	Standard Deviation
Replicate	1	2	3	1	2	3		
St. 1	ND	ND	ND	0.0	0.0	0.0	0.00	0.00
St. 2	ND	ND	ND	0.0	0.0	0.0	0.00	0.00
St. 3	ND	ND	36.06	0.0	0.0	1638.1	546.03	945.76
St. 4	36.13	37.07	36.06	1572.2	872.9	1645.7	1363.60	426.54
St. 5	ND	ND	ND	0.0	0.0	0.0	0.00	0.00
St. 6	ND	ND	ND	0.0	0.0	0.0	0.00	0.00
St. 7	ND	ND	ND	0.0	0.0	0.0	0.00	0.00
St. 8	ND	ND	ND	0.0	0.0	0.0	0.00	0.00
St. 9	ND	ND	ND	0.0	0.0	0.0	0.00	0.00
St. 10	ND	35.93	35.08	0.0	1787.2	3042.6	1609.93	1529.03
St. 11	ND	ND	ND	0.0	0.0	0.0	0.00	0.00
St. 12	34.95	ND	ND	3296.4	0.0	0.0	1098.80	1903.18
St. 13	34.68	ND	ND	3893.3	0.0	0.0	1297.77	2247.80

ND: not detected.

## Data Availability

The original contributions presented in this study are included in the article.

## Supplementary Materials

The following supporting information can be downloaded at: https://www.mdpi.com/article/10.3390/ani15233451/s1, Table S1: List of DNA sequences of the mitochondrially encoded cytochrome b gene of the bagrid species retrieved from the GenBank database of the National Center for Biotechnology Information; Data S1: Molecular Monitoring of Leiocassis longirostris by qPCR Assay.
