# Supplementary material for: Molecular Monitoring of Leiocassis longirostris Using Species-Specific qPCR Assays from Environmental DNA
Source: Animals (Basel). 2025 Nov 29;15(23):3451. doi: 10.3390/ani15233451 (PMC12691256; doi:10.3390/ani15233451)
Supplement: Supplementary file 1 [file animals-15-03451-s001.zip › Supplementary Table S1-Molecular Monitoring of Leiocassis longirostris by qPCR Assay-1st revision.docx]

**Supplementary Table S1.** List of DNA sequences of the mitochondrially encoded cytochrome *b* gene of the bagrid species retrieved from the GenBank database of the National Center for Biotechnology Information

| **Species** | **Specimen** | **GenBank accession number** | **Family** |
| --- | --- | --- | --- |
| *Ameiurus catus* | NEFC F16-216 | NC_037012 | Ictaluridae |
| *Ameiurus natalis* | NEFC_F16-116 | NC_036391 | Ictaluridae |
| *Ameiurus nebulosus* | NEFC_F16-113 | NC_042499 | Ictaluridae |
| *Arius arius* |  | NC_036673 | Ariidae |
| *Arius maculatus* |  | NC_045222 | Ariidae |
| *Bagre panamensis* |  | NC_037470 | Ariidae |
| *Hemibagrus guttatus* |  | NC_023976 | Bagridae |
| *Hemibagrus macropterus* |  | NC_019592 | Bagridae |
| *Hemibagrus nemurus* |  | NC_044863 | Bagridae |
| *Hemibagrus spilopterus* |  | NC_023222 | Bagridae |
| *Hemibagrus wyckioides* |  | NC_024278 | Bagridae |
| *Horabagrus brachysoma* |  | NC_030188 | Bagridae |
| *Ictalurus furcatus* |  | NC_028151 | Ictaluridae |
| *Ictalurus pricei* | TNHC-21704-10 | NC_029158 | Ictaluridae |
| *Ictalurus punctatus* | Norris | NC_003489 | Ictaluridae |
| *Kryptopterus bicirrhis* |  | NC_034999 | Siluridae |
| *Kryptopterus vitreolus* | 09 | NC_035419 | Siluridae |
| *Leiocassis longirostris* |  | NC_014586 | Bagridae |
| *Leiocassis longirostris* |  | MK458524 | Bagridae |
| *Leiocassis longirostris* |  | DQ321755 | Bagridae |
| *Leiocassis longirostris* | T020102MD | AY912440 | Bagridae |
| *Leiocassis longirostris* | IHB 0305228 | AY912441 | Bagridae |
| *Leiocassis longirostris* | IHB 0305229 | AY912442 | Bagridae |
| *Leiocassis longirostris* | IHB 20190805116 | MT330327 | Bagridae |
| *Liobagrus andersoni* | NIBRGR0000166819 | NC_032035 | Amblycipitidae |
| *Liobagrus anguillicauda* |  | NC_021602 | Amblycipitidae |
| *Liobagrus kingi* |  | NC_020337 | Amblycipitidae |
| *Liobagrus marginatus* |  | NC_022923 | Amblycipitidae |
| *Liobagrus mediadiposalis* |  | NC_027167 | Amblycipitidae |
| *Liobagrus nigricauda* |  | NC_021407 | Amblycipitidae |
| *Liobagrus obesus* |  | NC_008232 | Amblycipitidae |
| *Liobagrus somjinensis* |  | MN756661 | Amblycipitidae |
| *Liobagrus styani* | IHB2015111503 | NC_034647 | Amblycipitidae |
| *Mystus cavasius* |  | NC_030187 | Bagridae |
| *Mystus rhegma* |  | NC_023223 | Bagridae |
| *Mystus vittatus* |  | NC_032082 | Bagridae |
| *Netuma thalassina* |  | NC_030323 | Ariidae |
| *Noturus taylori* | LodgeLab Ntaylori_1 | NC_028276 | Ictaluridae |
| *Occidentarius platypogon* |  | NC_037469 | Ariidae |
| *Ompok bimaculatus* | OB-WM-TR01 | NC_036233 | Siluridae |
| *Ompok pabda* |  | NC_042212 | Siluridae |
| *Pelteobagrus eupogon* |  | NC_018768 | Bagridae |
| *Pelteobagrus vachellii* |  | NC_014862 | Bagridae |
| *Pseudobagrus albomarginatus* |  | NC_022726 | Bagridae |
| *Pseudobagrus brevicaudatus* |  | NC_021393 | Bagridae |
| *Pseudobagrus brevicorpus* | LEGO-F407 | NC_015625 | Bagridae |
| *Pseudobagrus brevicorpus* |  | HQ199229 | Bagridae |
| *Pseudobagrus emarginatus* |  | NC_024279 | Bagridae |
| *Pseudobagrus koreanus* |  | NC_028434 | Bagridae |
| *Pseudobagrus medianalis* |  | NC_037048 | Bagridae |
| *Pseudobagrus ondon* |  | NC_022725 | Bagridae |
| *Pseudobagrus pratti* |  | NC_041443 | Bagridae |
| *Pseudobagrus tenuis* |  | NC_035498 | Bagridae |
| *Pseudobagrus tokiensis* |  | NC_004697 | Bagridae |
| *Pseudobagrus trilineatus* |  | NC_022705 | Bagridae |
| *Pseudobagrus truncatus* |  | NC_021395 | Bagridae |
| *Pseudobagrus ussuriensis* |  | NC_020344 | Bagridae |
| *Pterocryptis cochinchinensis* |  | NC_027107 | Siluridae |
| *Pylodictis olivaris* | NEFC_F16-278 | NC_036386 | Ictaluridae |
| *Rita rita* |  | NC_023376 | Bagridae |
| *Silurus asotus* |  | NC_015806 | Siluridae |
| *Silurus glanis* |  | NC_014261 | Siluridae |
| *Silurus lanzhouensis* |  | NC_015650 | Siluridae |
| *Silurus meridionalis* |  | NC_014866 | Siluridae |
| *Silurus microdorsalis* |  | NC_028175 | Siluridae |
| *Silurus soldatovi* |  | NC_022723 | Siluridae |
| *Tachysurus argentivittatus* |  | NC_030538 | Bagridae |
| *Tachysurus crassilabris* |  | NC_021394 | Bagridae |
| *Tachysurus fulvidraco* |  | NC_015888 | Bagridae |
| *Tachysurus nitidus* |  | NC_014859 | Bagridae |
